# 4-Amino-3-(3-meth­oxy­benz­yl)-1*H*-1,2,4-triazole-5(4*H*)-thione

**DOI:** 10.1107/S1600536813009859

**Published:** 2013-04-13

**Authors:** B. K. Sarojini, P. S. Manjula, Gurumurthy Hegde, Dalbir Kour, Sumati Anthal, Vivek K. Gupta, Rajni Kant

**Affiliations:** a Department of Chemistry, P A College of Engineering, Nadupadavu 574 153, D.K. Mangalore, India; b Faculty of Industrial Science and Technology, University Malaysia Pahang, LebuhrayaTunRazak, 26300 Gambang, Kuantan, Pahang Darul Makmur, Malaysia; c X-ray Crystallography Laboratory, Post-Graduate Department of Physics & Electronics, University of Jammu, Jammu Tawi 180 006, India

## Abstract

In the title mol­ecule, C_10_H_12_N_4_SO, the triazole ring forms a dihedral angle of 73.0 (5)° with the benzene ring. The meth­oxy group is approximtely coplanar with the benzene ring with a C C—O—C_meth­yl_ torsion angle of 4.7 (3)°. In the crystal, N—H⋯S hydrogen bonds connect pairs of inversion-related mol­ecules, which are in turn connected by N—H⋯N hydrogen bonds into chains of rings along [010]. Weak C—H⋯O hydrogen bonds connect these chains into a two-dimensional network parallel to (-102).

## Related literature

For background to the chemistry of triazoles, see: Holla *et al.* (2001 ▶, 2006 ▶). For the biological activity of 1,2,4-triazole derivatives, see: Cansiz *et al.* (2001 ▶); Jones *et al.* (1965 ▶); Kane *et al.* (1988 ▶); Mullican *et al.* (1993 ▶). For related structures, see: Chen *et al.* (2007 ▶); Gao *et al.* (2011 ▶); Karczmarzyk *et al.* (2012 ▶). For standard bond-length data, see: Allen *et al.* (1987 ▶).
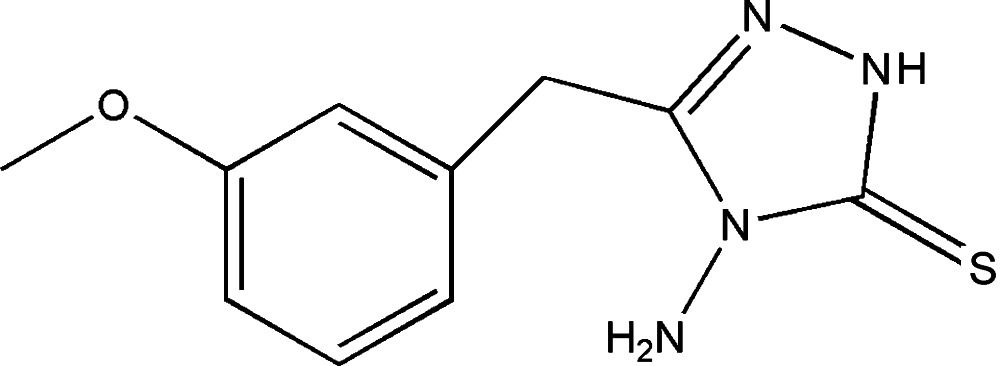


## Experimental

### 

#### Crystal data

C_10_H_12_N_4_OS
*M* *_r_* = 236.30
Monoclinic, 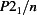

*a* = 7.4580 (3) Å
*b* = 5.8006 (2) Å
*c* = 25.2817 (10) Å
β = 94.513 (4)°
*V* = 1090.32 (7) Å^3^
*Z* = 4
Mo *K*α radiation
μ = 0.28 mm^−1^
*T* = 293 K
0.3 × 0.2 × 0.1 mm

#### Data collection

Oxford Diffraction Xcalibur Sapphire3 diffractometer
Absorption correction: multi-scan (*CrysAlis PRO*; Oxford Diffraction, 2010 ▶) *T*_min_ = 0.946, *T*_max_ = 1.000
15190 measured reflections
2130 independent reflections
1748 reflections with *I* > 2σ(*I*)
*R*_int_ = 0.042

#### Refinement

*R*[*F* ^2^ > 2σ(*F* ^2^)] = 0.036
*wR*(*F* ^2^) = 0.088
*S* = 1.03
2130 reflections
154 parameters
H atoms treated by a mixture of independent and constrained refinement
Δρ_max_ = 0.19 e Å^−3^
Δρ_min_ = −0.24 e Å^−3^

### 

Data collection: *CrysAlis PRO* (Oxford Diffraction, 2010 ▶); cell refinement: *CrysAlis PRO*; data reduction: *CrysAlis PRO*; program(s) used to solve structure: *SHELXS97* (Sheldrick, 2008 ▶); program(s) used to refine structure: *SHELXL97* (Sheldrick, 2008 ▶); molecular graphics: *PLATON* (Spek, 2009 ▶); software used to prepare material for publication: *PLATON*.

## Supplementary Material

Crystal structure: contains datablock(s) I, global. DOI: 10.1107/S1600536813009859/lh5605sup1.cif

Structure factors: contains datablock(s) I. DOI: 10.1107/S1600536813009859/lh5605Isup2.hkl

Supplementary material file. DOI: 10.1107/S1600536813009859/lh5605Isup3.cml

Additional supplementary materials:  crystallographic information; 3D view; checkCIF report

## Figures and Tables

**Table 1:** Hydrogen-bond geometry (Å, °)

*D*—H⋯*A*	*D*—H	H⋯*A*	*D*⋯*A*	*D*—H⋯*A*
C7—H7*A*⋯O1^i^	0.97	2.46	3.308 (2)	146
N6—H62⋯N1^ii^	0.90 (2)	2.30 (2)	3.190 (2)	174
N2—H2⋯S1^iii^	0.86	2.60	3.377 (1)	151

Symmetry codes: (i) 
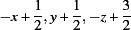
; (ii) 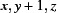; (iii) 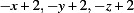.

**Fig. 1.**
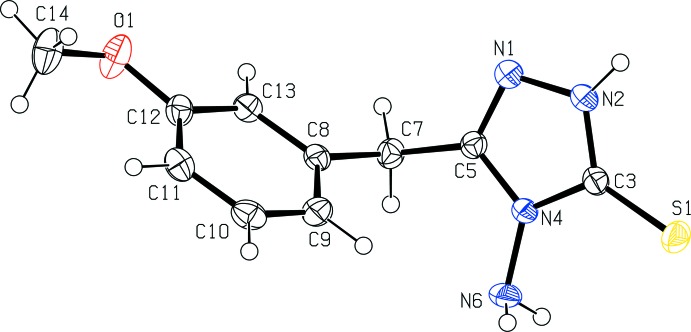
The molecular structure of the title compound with ellipsoids drawn at the 40% probability level. H atoms are shown as small spheres of arbitrary radii.

**Fig. 2.**
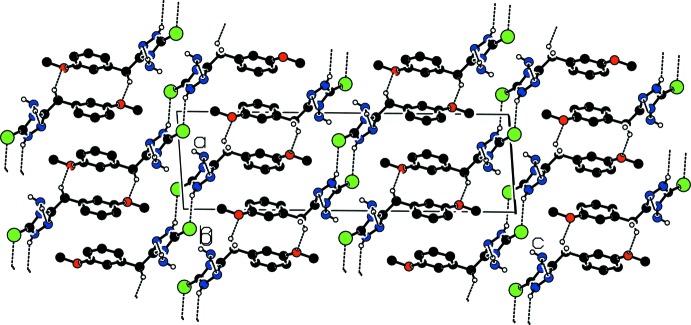
Part of the crystal structure with hydrogen bonds shown as dashed lines.

## supplementary crystallographic information

### Comment

The chemistry of triazoles has received considerable attention in recent years because of their versatility in the synthesis of many other heterocyclic compounds. 1,2,4-Triazole derivatives are well known for their different biological activities, therefore various 1,2,4-triazole derivatives and their N-bridged heterocyclic analogs have been extensively studied (Holla *et al.*, 2001; 2006). The derivatives of 1,2,4-triazole are known to exhibit anti-inflammatory (Mullican *et al.*, 1993), antiviral (Jones *et al.*, 1965), antimicrobial (Cansiz *et al.*, 2001), and antidepressant activity (Kane *et al.*, 1988). Hence, synthesis of the corresponding heterocyclic compounds could be of interest from the viewpoint of chemical reactivity and biological activity. In the title compound (Fig. 1), the bond lengths and angles have normal values (Allen *et al.*, 1987) and are comparable with closely related structures (Chen *et al.*, 2007; Karczmarzyk *et al.*, 2012; Gao *et al.*, 2011). The angles around atom C3 in the triazole ring deviate from the normal angles based on C*sp*^2^ hybridization, giving bond angles of 102.72 (14)° and 130.12 (13)° for N2—C3—N4 and N2—C3—S1, respectively. The dihedral angle between the triazole ring (N1/N2/C3/N4/C5) and the benzene ring (C8—C13) is 73.0 (5)°. In the crystal, N—H···S hydrogen bonds connect pairs of inversion related molecules, which are in turn connected by N—H···N hydrogen bonds into chains of rings along [010]. In addition, weak C—H···O hydrogen bonds connect these chains into a two-dimensional network parallel to (102) (Fig. 2).

### Experimental

A well triturated mixture of 3-methoxyphenylacetic acid (0.83 g.0.05 mol) and thiocarbohydrazide (0.53 g. 0.05 mol) was fused in a round bottom flask for one hour on a oil bath at 413 K. It was cooled to room temperature and washed with sodium bicarbonate (5%) solution to remove unreacted acid and again washed with water. The dried compound was recrystallized from methanol to yield single crystals (mp. 417–419 K).

### Refinement

Atoms H61 and H62 attached to N6 were located in a difference map and refined isotropically. The remaining H atoms were positioned geometrically and were treated as riding on their parent C/N atoms, with C—H distances of 0.93–0.96 Å and N—H distance of 0.86 Å with *U*_iso_(H) = 1.2*U*_eq_(C/N) or *U*_iso_(H) = 1.5*U*_eq_(C_methyl_).

### Crystal data

**Table:**

C_10_H_12_N_4_OS	*F*(000) = 496
*M**_r_* = 236.30	*D*_x_ = 1.440 Mg m^−^^3^
Monoclinic, *P*2_1_/*n*	Mo *K*α radiation, λ = 0.71073 Å
Hall symbol: -P 2yn	Cell parameters from 7374 reflections
*a* = 7.4580 (3) Å	θ = 3.5–29.0°
*b* = 5.8006 (2) Å	µ = 0.28 mm^−^^1^
*c* = 25.2817 (10) Å	*T* = 293 K
β = 94.513 (4)°	Plate, white
*V* = 1090.32 (7) Å^3^	0.3 × 0.2 × 0.1 mm
*Z* = 4	

### Data collection

**Table:**

Oxford Diffraction Xcalibur Sapphire3 diffractometer	2130 independent reflections
Radiation source: fine-focus sealed tube	1748 reflections with *I* > 2σ(*I*)
Graphite monochromator	*R*_int_ = 0.042
Detector resolution: 16.1049 pixels mm^-1^	θ_max_ = 26.0°, θ_min_ = 3.5°
ω scans	*h* = −9→9
Absorption correction: multi-scan (*CrysAlis PRO*; Oxford Diffraction, 2010)	*k* = −7→7
*T*_min_ = 0.946, *T*_max_ = 1.000	*l* = −31→31
15190 measured reflections	

### Refinement

**Table:**

Refinement on *F*^2^	Primary atom site location: structure-invariant direct methods
Least-squares matrix: full	Secondary atom site location: difference Fourier map
*R*[*F*^2^ > 2σ(*F*^2^)] = 0.036	Hydrogen site location: inferred from neighbouring sites
*wR*(*F*^2^) = 0.088	H atoms treated by a mixture of independent and constrained refinement
*S* = 1.03	*w* = 1/[σ^2^(*F*_o_^2^) + (0.0408*P*)^2^ + 0.3289*P*] where *P* = (*F*_o_^2^ + 2*F*_c_^2^)/3
2130 reflections	(Δ/σ)_max_ = 0.002
154 parameters	Δρ_max_ = 0.19 e Å^−^^3^
0 restraints	Δρ_min_ = −0.24 e Å^−^^3^

### Special details

**Table:**

Experimental. *CrysAlis PRO*, Oxford Diffraction Ltd., Version 1.171.34.40 (release 27–08-2010 CrysAlis171. NET) (compiled Aug 27 2010,11:50:40) Empirical absorption correction using spherical harmonics, implemented in SCALE3 ABSPACK scaling algorithm.
Geometry. All e.s.d.'s (except the e.s.d. in the dihedral angle between two l.s. planes) are estimated using the full covariance matrix. The cell e.s.d.'s are taken into account individually in the estimation of e.s.d.'s in distances, angles and torsion angles; correlations between e.s.d.'s in cell parameters are only used when they are defined by crystal symmetry. An approximate (isotropic) treatment of cell e.s.d.'s is used for estimating e.s.d.'s involving l.s. planes.
Refinement. Refinement of *F*^2^ against ALL reflections. The weighted *R*-factor *wR* and goodness of fit *S* are based on *F*^2^, conventional *R*-factors *R* are based on *F*, with *F* set to zero for negative *F*^2^. The threshold expression of *F*^2^ > σ(*F*^2^) is used only for calculating *R*-factors(gt) *etc*. and is not relevant to the choice of reflections for refinement. *R*-factors based on *F*^2^ are statistically about twice as large as those based on *F*, and *R*- factors based on ALL data will be even larger.

### Fractional atomic coordinates and isotropic or equivalent isotropic displacement parameters (Å^2^)

**Table:**

	*x*	*y*	*z*	*U*_iso_*/*U*_eq_	
S1	0.79363 (6)	1.26508 (8)	1.014843 (19)	0.03712 (16)	
O1	0.4364 (2)	0.7659 (2)	0.66746 (5)	0.0467 (4)	
N1	0.6715 (2)	0.7764 (2)	0.91172 (6)	0.0323 (4)	
N2	0.77124 (19)	0.8746 (2)	0.95420 (6)	0.0319 (4)	
H2	0.8621	0.8077	0.9707	0.038*	
C3	0.7134 (2)	1.0833 (3)	0.96726 (6)	0.0254 (4)	
N4	0.56824 (17)	1.1164 (2)	0.93177 (5)	0.0239 (3)	
C5	0.5471 (2)	0.9287 (3)	0.89877 (6)	0.0246 (4)	
N6	0.4558 (2)	1.3106 (3)	0.92853 (8)	0.0360 (4)	
C7	0.4041 (2)	0.9044 (3)	0.85492 (6)	0.0284 (4)	
H7A	0.2994	0.9908	0.8639	0.034*	
H7B	0.3696	0.7434	0.8519	0.034*	
C8	0.4584 (2)	0.9870 (3)	0.80138 (6)	0.0249 (4)	
C9	0.5382 (2)	1.2018 (3)	0.79529 (7)	0.0322 (4)	
H9	0.5617	1.2972	0.8246	0.039*	
C10	0.5823 (2)	1.2730 (3)	0.74597 (8)	0.0339 (4)	
H10	0.6347	1.4170	0.7423	0.041*	
C11	0.5501 (2)	1.1344 (3)	0.70177 (7)	0.0328 (4)	
H11	0.5805	1.1838	0.6686	0.039*	
C12	0.4717 (2)	0.9206 (3)	0.70779 (7)	0.0290 (4)	
C13	0.4246 (2)	0.8490 (3)	0.75726 (7)	0.0271 (4)	
H13	0.3696	0.7064	0.7607	0.032*	
C14	0.4954 (3)	0.8208 (4)	0.61719 (8)	0.0492 (5)	
H14A	0.4348	0.9572	0.6037	0.074*	
H14B	0.4686	0.6950	0.5932	0.074*	
H14C	0.6228	0.8475	0.6205	0.074*	
H62	0.524 (3)	1.436 (4)	0.9246 (8)	0.049 (6)*	
H61	0.403 (3)	1.324 (4)	0.9573 (10)	0.065 (8)*	

### Atomic displacement parameters (Å^2^)

**Table:**

	*U*^11^	*U*^22^	*U*^33^	*U*^12^	*U*^13^	*U*^23^
S1	0.0330 (3)	0.0444 (3)	0.0325 (3)	0.0079 (2)	−0.00694 (19)	−0.0124 (2)
O1	0.0607 (9)	0.0517 (8)	0.0284 (7)	−0.0206 (7)	0.0078 (6)	−0.0085 (6)
N1	0.0352 (8)	0.0312 (8)	0.0297 (9)	0.0065 (6)	−0.0034 (7)	−0.0030 (6)
N2	0.0317 (8)	0.0324 (8)	0.0303 (8)	0.0109 (6)	−0.0064 (7)	−0.0018 (6)
C3	0.0232 (8)	0.0305 (9)	0.0227 (9)	0.0032 (7)	0.0025 (7)	0.0025 (7)
N4	0.0223 (7)	0.0239 (7)	0.0250 (7)	0.0044 (5)	−0.0015 (6)	−0.0009 (6)
C5	0.0267 (8)	0.0244 (8)	0.0228 (9)	−0.0007 (7)	0.0033 (7)	0.0004 (6)
N6	0.0353 (9)	0.0290 (9)	0.0422 (11)	0.0141 (7)	−0.0073 (8)	−0.0061 (7)
C7	0.0264 (9)	0.0310 (9)	0.0273 (9)	−0.0015 (7)	−0.0010 (7)	−0.0032 (7)
C8	0.0204 (8)	0.0265 (8)	0.0272 (9)	0.0033 (6)	−0.0028 (7)	0.0003 (7)
C9	0.0355 (10)	0.0263 (9)	0.0341 (10)	−0.0017 (7)	−0.0023 (8)	−0.0043 (7)
C10	0.0326 (10)	0.0238 (9)	0.0450 (11)	−0.0031 (7)	0.0003 (8)	0.0055 (8)
C11	0.0311 (9)	0.0363 (10)	0.0310 (10)	−0.0006 (8)	0.0016 (8)	0.0079 (8)
C12	0.0261 (9)	0.0335 (9)	0.0267 (9)	−0.0010 (7)	−0.0012 (7)	−0.0008 (7)
C13	0.0256 (9)	0.0248 (8)	0.0304 (10)	−0.0024 (7)	−0.0004 (7)	0.0008 (7)
C14	0.0469 (13)	0.0726 (15)	0.0287 (11)	−0.0096 (11)	0.0073 (9)	−0.0059 (10)

### Geometric parameters (Å, º)

**Table:**

S1—C3	1.6745 (17)	C7—H7B	0.9700
O1—C12	1.368 (2)	C8—C13	1.380 (2)
O1—C14	1.414 (2)	C8—C9	1.395 (2)
N1—C5	1.304 (2)	C9—C10	1.377 (3)
N1—N2	1.381 (2)	C9—H9	0.9300
N2—C3	1.335 (2)	C10—C11	1.382 (3)
N2—H2	0.8600	C10—H10	0.9300
C3—N4	1.364 (2)	C11—C12	1.384 (2)
N4—C5	1.373 (2)	C11—H11	0.9300
N4—N6	1.4029 (19)	C12—C13	1.389 (2)
C5—C7	1.483 (2)	C13—H13	0.9300
N6—H62	0.90 (2)	C14—H14A	0.9600
N6—H61	0.86 (3)	C14—H14B	0.9600
C7—C8	1.521 (2)	C14—H14C	0.9600
C7—H7A	0.9700		
			
C12—O1—C14	117.84 (15)	C13—C8—C7	119.48 (15)
C5—N1—N2	104.16 (13)	C9—C8—C7	121.71 (15)
C3—N2—N1	113.69 (14)	C10—C9—C8	120.12 (16)
C3—N2—H2	123.2	C10—C9—H9	119.9
N1—N2—H2	123.2	C8—C9—H9	119.9
N2—C3—N4	102.72 (14)	C9—C10—C11	121.26 (16)
N2—C3—S1	130.12 (13)	C9—C10—H10	119.4
N4—C3—S1	127.17 (12)	C11—C10—H10	119.4
C3—N4—C5	109.59 (13)	C10—C11—C12	118.69 (17)
C3—N4—N6	126.24 (14)	C10—C11—H11	120.7
C5—N4—N6	124.17 (14)	C12—C11—H11	120.7
N1—C5—N4	109.83 (15)	O1—C12—C11	124.32 (16)
N1—C5—C7	125.34 (15)	O1—C12—C13	115.29 (15)
N4—C5—C7	124.83 (14)	C11—C12—C13	120.39 (16)
N4—N6—H62	108.4 (14)	C8—C13—C12	120.74 (15)
N4—N6—H61	109.5 (17)	C8—C13—H13	119.6
H62—N6—H61	109 (2)	C12—C13—H13	119.6
C5—C7—C8	114.15 (14)	O1—C14—H14A	109.5
C5—C7—H7A	108.7	O1—C14—H14B	109.5
C8—C7—H7A	108.7	H14A—C14—H14B	109.5
C5—C7—H7B	108.7	O1—C14—H14C	109.5
C8—C7—H7B	108.7	H14A—C14—H14C	109.5
H7A—C7—H7B	107.6	H14B—C14—H14C	109.5
C13—C8—C9	118.79 (16)		
			
C5—N1—N2—C3	−0.81 (19)	C5—C7—C8—C13	132.14 (16)
N1—N2—C3—N4	1.00 (18)	C5—C7—C8—C9	−49.4 (2)
N1—N2—C3—S1	−179.11 (13)	C13—C8—C9—C10	−0.1 (3)
N2—C3—N4—C5	−0.80 (17)	C7—C8—C9—C10	−178.66 (16)
S1—C3—N4—C5	179.30 (13)	C8—C9—C10—C11	−0.4 (3)
N2—C3—N4—N6	179.29 (16)	C9—C10—C11—C12	0.1 (3)
S1—C3—N4—N6	−0.6 (3)	C14—O1—C12—C11	4.7 (3)
N2—N1—C5—N4	0.25 (18)	C14—O1—C12—C13	−174.70 (17)
N2—N1—C5—C7	−179.94 (15)	C10—C11—C12—O1	−178.59 (17)
C3—N4—C5—N1	0.36 (19)	C10—C11—C12—C13	0.8 (3)
N6—N4—C5—N1	−179.74 (16)	C9—C8—C13—C12	1.0 (2)
C3—N4—C5—C7	−179.46 (15)	C7—C8—C13—C12	179.59 (15)
N6—N4—C5—C7	0.4 (3)	O1—C12—C13—C8	178.05 (15)
N1—C5—C7—C8	−87.4 (2)	C11—C12—C13—C8	−1.4 (3)
N4—C5—C7—C8	92.39 (19)		

### Hydrogen-bond geometry (Å, º)

**Table:**

*D*—H···*A*	*D*—H	H···*A*	*D*···*A*	*D*—H···*A*
C7—H7*A*···O1^i^	0.97	2.46	3.308 (2)	146
N6—H62···N1^ii^	0.90 (2)	2.30 (2)	3.190 (2)	174
N2—H2···S1^iii^	0.86	2.60	3.377 (1)	151

Symmetry codes: (i) −*x*+1/2, *y*+1/2, −*z*+3/2; (ii) *x*, *y*+1, *z*; (iii) −*x*+2, −*y*+2, −*z*+2.
